# ATZ‐1 promotes DNA replication efficiency to maintain normal meiotic function

**DOI:** 10.1002/2211-5463.70300

**Published:** 2026-07-09

**Authors:** Taylin E. Gourley, Luke W. Molesworth, Nethma H. Waduge, Fahimeh Eskandari, Peter R. Boag, Gregory M. Davis

**Affiliations:** ^1^ Federation University Institute of Innovation, Science and Sustainability Australia; ^2^ Department of Biomedical, Health and Exercise Sciences Swinburne University of Technology Hawthorn Victoria Australia; ^3^ Department of Biochemistry and Molecular Biology Monash University Clayton Australia

**Keywords:** cell cycle, DNA replication, germ cells, meiosis, mitosis, oogenesis

## Abstract

Precise coordination of DNA replication and meiotic progression is essential for germline genome stability, yet a complete understanding of this interplay remains unknown. In this study, we show that the absence of Abnormal Transition Zone 1 (*atz‐1*) in *Caenorhabditis elegans* results in abnormal germline architecture and oocyte development. Specifically, *atz‐1* mutants display depleted S‐phase DNA replication and acute sensitivity to depletion of the checkpoint kinase 1 (*chk‐1*), but not *chk‐2*. These findings suggest that ATZ‐1 promotes replication efficiency during premeiotic S‐phase, potentially suppressing replication stress and enabling timely meiotic progression. Collectively, this work demonstrates that ATZ‐1 contributes to germline genome integrity by influencing cell cycle processes, which, if disrupted, result in numerous downstream germline defects.

## Graphical abstract

Absence of ATZ‐1 interferes with meiotic DNA replication and cell cycle function via CHK‐1. This causes downstream defects associated with DNA damage and genomic integrity. Taken together, this study suggests that ATZ‐1 influences DNA replication efficiency and cell cycle function to maintain normal meiotic function.

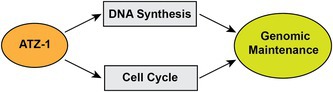

**Abbreviations**
ATM: Ataxia Telangiectasia Mutated
ATR: ATM and Rad3‐related
ATZ‐1: Abnormal Transition Zone 1
CDK: Cyclin‐Dependent Kinase
CHK‐1: Checkpoint Kinase 1
CHK‐2: Checkpoint Kinase 2
DAPI: 4′,6‐diamidino‐2‐phenylindole
DNA: Deoxyribonucleic acid
Edu: 5‐ethynyl‐2′‐deoxyuridine
NGM: Nutrient Growth Media
REC‐8: RECombination abnormal 8
RNAi: RNA Interference

DNA damage repair mechanisms and the cell cycle are tightly coordinated processes that ensure genomic integrity is preserved across successive rounds of cell division. This requires integration of DNA damage signalling, repair pathway choice, and checkpoint control [1, 2]. Central to these processes are the checkpoint kinases ataxia telangiectasia mutated (ATM) and ATM and Rad3‐related (ATR), which are activated in response to double‐strand DNA breaks and replication stress [3, 4]. Upon activation, ATM and ATR phosphorylate downstream effectors such as CHK2 and CHK1, which inhibit cyclin‐dependent kinase (CDK) activity through stabilisation of CDK inhibitors or degradation of CDC25 phosphatases [5, 6] This results in cell‐cycle arrest at key transitions including G1/S, intra‐S, and G2/M, allowing time for the DNA damage repair machinery to facilitate repair [7].

Although present in most cell types, germ cells are heavily reliant on these mechanisms, where errors during early meiotic events can lead to defective gametogenesis and the proliferation of heritable mutations [8, 9]. In *Caenorhabditis elegans*, cell‐cycle checkpoints are largely mediated by the kinase proteins CHK‐1 and CHK‐2. While both contribute to maintaining genomic integrity, CHK‐1 is primarily associated with S‐phase processes including DNA replication and replication fork stability, whereas CHK‐2 functions predominantly during meiotic prophase and homologous chromosome pairing [10, 11, 12]. These kinases act in overlapping and partially redundant pathways to ensure that cell‐cycle progression and meiotic entry are tightly regulated [11, 13].

The germ cell transition zone protein Abnormal Transition Zone 1 (ATZ‐1) has previously been shown to influence germ cell DNA replication and the loading of the cohesin protein REC‐8 onto sister chromatids [14]. Loss of *atz‐1* results in abnormal chromosome morphology in oocytes and increased sensitivity to the DNA replication inhibitor, hydroxyurea. Although structural modelling initially suggested that ATZ‐1 may resemble a myosin heavy chain protein, bioinformatic analyses indicate that it lacks key myosin‐specific domains [15]. In addition to this, ATZ‐1 associates with proteins involved in transcription and the mitosis‐to‐meiosis transition, including CCAR‐1 and MEMI‐1, suggesting a broader role in early germline development and meiotic regulation [16, 17].

In this study, we hypothesised that ATZ‐1 contributes to early cell‐cycle events in the germline, particularly during premeiotic S‐phase. We show that loss of *atz‐1* results in defects in DNA synthesis, altered meiotic timing, and abnormalities in oocyte and sperm organisation. Furthermore, *atz‐1* mutants display enhanced sensitivity to *chk‐1* knockdown but not *chk‐2*, indicating a functional interaction with S‐phase checkpoint pathways. Together, these findings suggest that ATZ‐1 plays an important role in coordinating DNA replication with downstream meiotic processes.

## Materials and methods

### Maintenance of strains

This study used the N2 as the wild‐type control, and the *atz‐1(ok3406)* deletion strain was used for all experiments and duplicated with RNAi where mentioned for confirmation. Strains were maintained at 20 °C and 25 °C under standard conditions [18]. The *atz‐1(ok3406)* strain was backcrossed five times prior to analysis. Genotyping was performed using the following primers: outer forward (CCTCTTCTCCCTCCTCATCA), outer reverse (TCATTTCACTCGCACAGGTC), inner forward (TTCAACCTCGTAGTTCTCCTCC), and inner reverse (CGACTGGTTGTCACCCTTTT).

### Germline analysis and DAPI staining

Germline dissections and DAPI staining were performed as previously described [19]. Briefly, Day 1 adult animals were anaesthetised in 0.001% tetramisole in M9 buffer on coverslips and dissected using syringe needles to isolate germlines. Samples were transferred to poly‐L‐lysine‐coated slides and snap‐frozen on dry ice. Slides were washed twice for 5 min in PBS containing 0.5% Tween‐20 (PBST), then incubated with DAPI (1 : 1000 dilution in normal goat serum) for 1 h at room temperature. After washing twice in PBST, slides were mounted using Dako Fluorescent Mounting Medium (Dako, Denmark). Germline analysis was performed in triplicates (*N* = 30 per replicate). Images were acquired using a Zeiss Axiolab microscope equipped with a pE‐300 LED fluorescence system and a Tucsen monochrome CCD camera (Tuscen, China).

### RNA interference

RNAi clones were obtained from the ORFeome library [20] and delivered by feeding as previously described [21]. Briefly, bacterial cultures were grown overnight in 2 × TY medium containing 100 mg/mL ampicillin at 37 °C, then seeded onto NGM plates supplemented with 4 mm IPTG and 100 mg/mL ampicillin. L1‐stage animals were transferred to RNAi plates and maintained at 20 °C or 25 °C until Day 1 adulthood prior to analysis.

### Brood size assay

Animals were maintained at 20 °C or 25 °C until the L4 stage, then transferred individually to seeded NGM plates. Animals were moved to fresh plates every 12 h until egg laying ceased. Plates were incubated at the respective temperatures and scored after 48 h. Total brood size was calculated as the number of progeny, including unhatched embryos. Embryonic lethality was expressed as a percentage of total progeny. Experiments were performed in triplicate (*N* = 10 animals per replicate).

### EdU assay

DNA synthesis was assessed using 5′‐ethynyl‐2′‐deoxyuridine (EdU) incorporation with a Click‐iT imaging kit (Invitrogen, USA). Animals were washed in M9 buffer and incubated in 20 μm EdU for 30 min with gentle rotation. Following incubation, animals were transferred to NGM plates, and Day 1 adults were dissected and fixed in −20 °C methanol. Slides were incubated with Click‐iT reaction cocktail for 30 min in the dark, washed twice in PBST for 10 min, and counterstained with DAPI. Samples were imaged using fluorescence microscopy. This was performed in triplicates (*N* = 30 per replicate).

### Statistical analysis

All assays were conducted in triplicates, then graphed and analysed for statistical significance via a Student's *t*‐test using the Prism5 software package (graphpad Software, USA), and images were processed using Adobe Photoshop and Illustrator (Adobe Systems, USA).

## Results

### *atz‐1* influences normal oocyte maturation

**Fig. 1:**
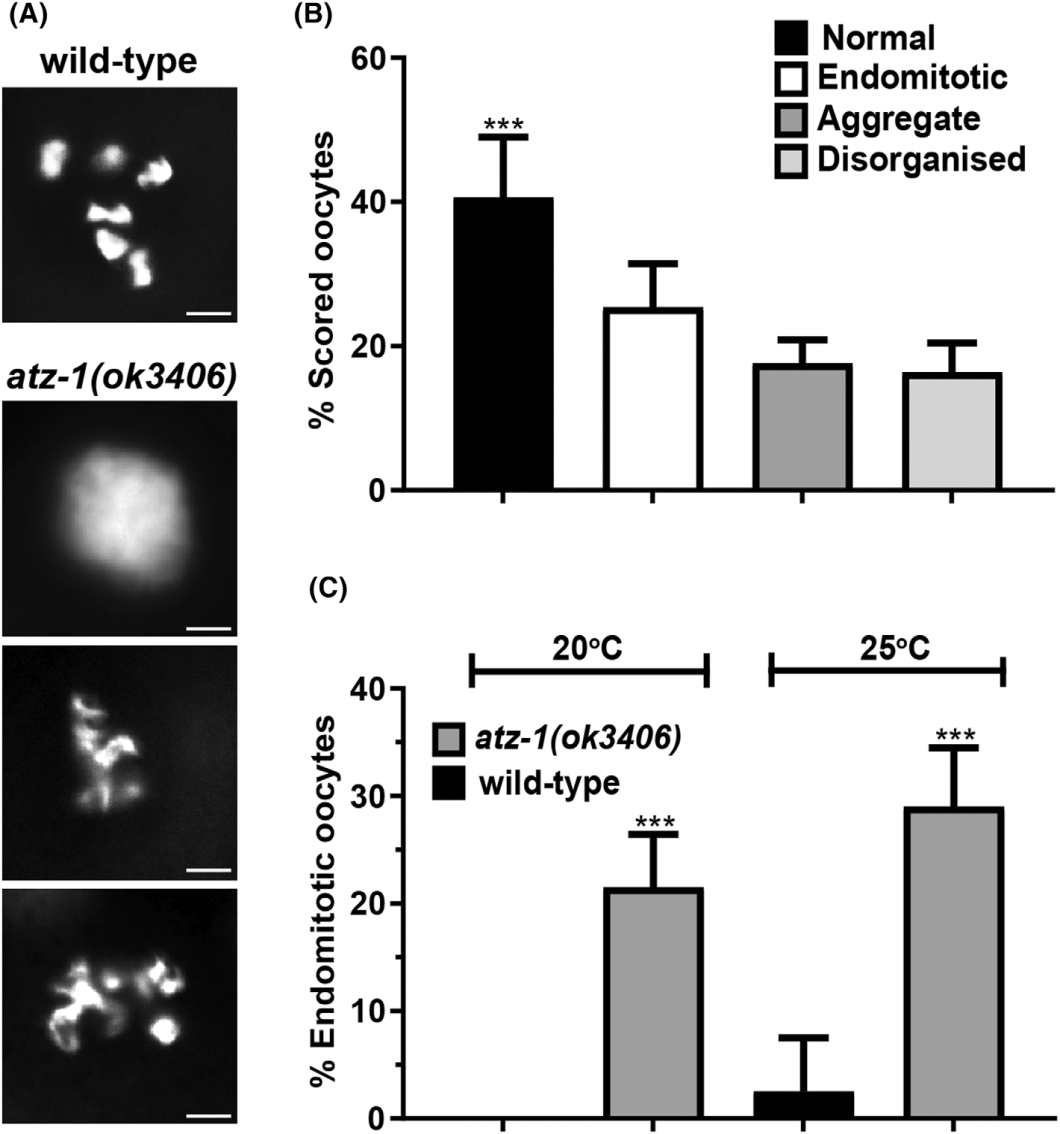
Absence of *atz‐1* results in endomitotic oocytes and disorganised chromosomes: (A) Representative images of diakinetic oocytes in wild‐type animals and *atz‐1(ok3406)* mutants. Wild‐type oocyte chromosomes display six bivalent chromosomal bodies (top), while *atz‐1(ok3406)* mutants display endomitotic oocytes or oocytes with aggregated or disorganised chromosomal bodies. Scale bar = 5 μm (B) Percentage of germlines in *atz‐1(ok3406)* mutants with abnormal chromosome morphology at 25 °C. *N* = 90. Error bars represent standard error of mean (SEM), ***P < 0.001. (C) Percentage of germlines with endomitotic oocytes in *atz‐1(ok3406)* mutants and wild‐type animals at 20 °C and 25 °C. *N* = 30. Error bars represent SEM. ***P < 0.001.

Oocyte maturation is reliant on multiple coordinated pathways and is typically associated with the transition from diakinesis to metaphase I [22]. Excessive ploidy in oocytes is commonly linked to prolonged exposure to sperm, ovulation delay, or defects in cell‐cycle regulation and DNA repair, resulting in endomitotic oocytes [23, 24, 25]. We previously reported that loss of *atz‐1* results in abnormal chromosome morphology and defects associated with meiosis I [14]. To investigate this further, germlines from gravid adults were dissected and the morphology of diakinetic oocytes was assessed in greater detail. As expected, *atz‐1(ok3406)* mutants displayed aggregated and disorganised chromosomes compared to wild‐type animals (Fig. 1A,B). In addition to this, *atz‐1(ok3406)* mutants exhibited a significant increase in endomitotic oocytes (Fig. 1A,C). This phenotype was observed in day one adults, indicating that it is not simply age‐dependent. Notably, the frequency of endomitotic oocytes was higher at 20 °C compared to 25 °C, suggesting that the defect is robust and not strongly temperature‐dependent. Additionally, this was phenocopied in *atz‐1(RNAi)* animals, suggesting that this observation is not allele‐specific (Fig. S1A). These findings, together with our previous observations of reduced germline progression from the mitotic region into meiosis I, are consistent with a defect in cell‐cycle timing in *atz‐1(ok3406)* mutants.

### *atz‐1* is required for genomic timing and oocyte/sperm specification and normal meiotic timing

**Fig. 2:**
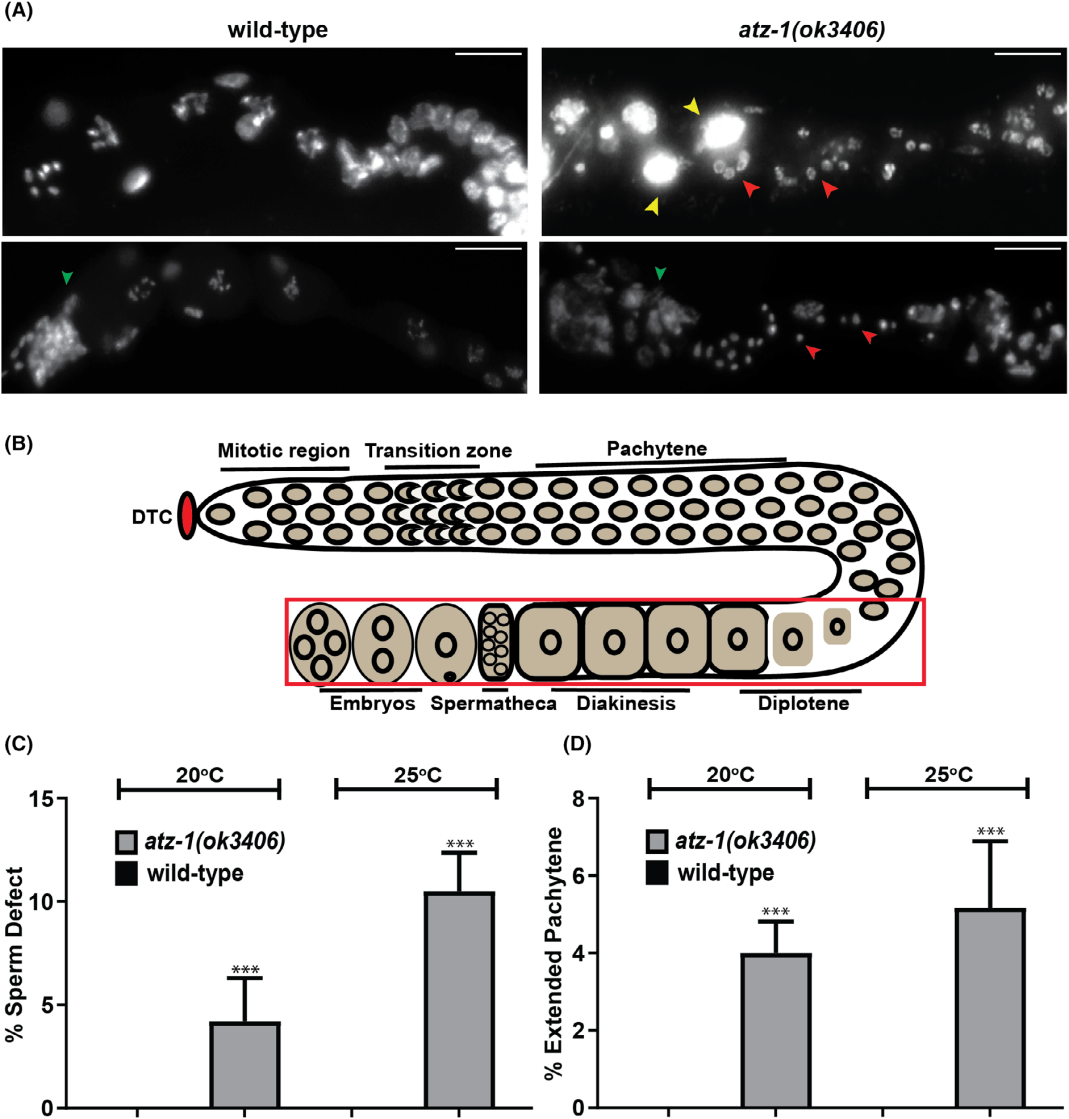
*atz‐1* is required for normal meiotic timing: (A) Representative images of wild‐type and *atz‐1(ok3406)* germlines at 25 °C. Wild‐type germlines exhibit a clear progression of germ cells through diakinesis before reaching the spermatheca (green arrow). *atz‐1(ok3406)* germlines display both endomitotic oocytes (yellow arrows) and pachytene stage germ cells extending into the diplotene and diakinetic region (top right, red arrows), and sperm (bottom right, red arrows) that distally escape the spermatheca. Scale bar = 50 μm (B) Illustration of oogenesis in *C. elegans* hermaphrodites with the region outlined in A. (C,D) Percentage distribution of wild‐type and *atz‐1(ok3406)* germlines scored for the sperm containment defect and the extended pachytene defect in wild‐type and *atz‐1(ok3406)* at 20 °C and 25 °C. *n* = 90. Error bars represent SEM. ***P < 0.001.

Spermatogenesis in *C. elegans* hermaphrodites occurs during the L4 larval stage, producing a defined number of sperm that are subsequently stored within the spermatheca [26, 27] (Fig. 2B). In wild‐type animals, sperm are retained within the spermatheca and prevented from moving distally by a constriction at its proximal boundary [25, 28]. In contrast to this, deletion or depletion of *atz‐1* results in a sperm containment defect, where sperm were observed outside of the spermatheca and within the proximal germline (Figs 2A,C and S1B). This phenotype is relatively rare and has been shown to be associated with defects in germline organisation or signalling [29, 30]. Furthermore, the presence of ectopic sperm may also contribute to the increased frequency of endomitotic oocytes, as sperm‐derived signals can induce endomitosis in oocytes [24, 31].

In addition to this defect, *atz‐1(ok3406)* mutants and *atz‐1(RNAi)* animals exhibited an extension of the pachytene region beyond its normal boundary (Figs 2A,D and S1C). In wild‐type animals, the pachytene region is restricted to a defined segment of the gonad arm, whereas in *atz‐1* mutants, pachytene nuclei frequently extended into diplotene and diakinesis regions. This phenotype was not observed in wild‐type germlines and is consistent with a delay in meiotic progression. Together, these findings suggest that loss of *atz‐1* disrupts normal germline organisation and meiotic timing, potentially due to defects in cell‐cycle progression or checkpoint activation.

### *atz‐1(ok3406)* worms exhibit genomic synthesis dysfunction and synthetically interact with *chk‐1*

**Fig. 3:**
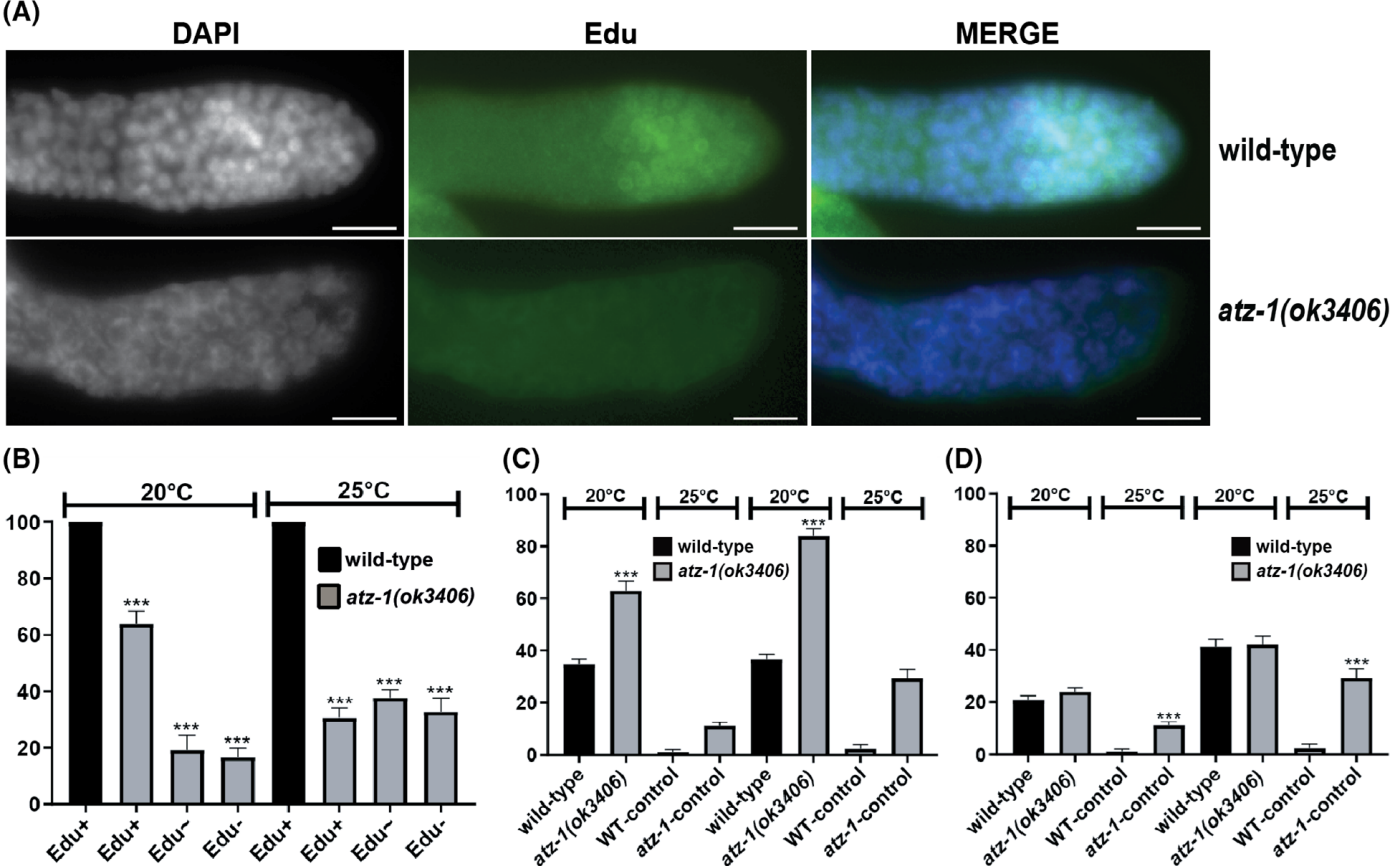
*atz‐1* is required for optimal genomic synthesis in pre‐meiotic S‐phase. (A) Mitotic zones of wild‐type (top) and *atz‐1(ok3406)* (bottom) germlines were stained with Edu for active DNA synthesis. Distal tips of *atz‐1(ok3406)* mutants display reduced levels of active S‐phase replication compared to wild‐type. Scale bar = 30 μm (B) Quantification of germlines scored for normal Edu distribution (+), partial (~) or no Edu distribution (−). Error bars represent SEM, *N* = 60 ***P < 0.001. (C,D) Percentage of embryonic lethality of (C) *chk‐1(RNAi)* and (D) *chk‐2(RNAi)* showing enhanced embryonic lethality when *chk‐1* was knocked down in *atz‐1* mutants compared to wild‐type animals with little change in *chk‐2* knockdown. Error bars represent SEM. *N* = 30 ***P < 0.001.

Accurate DNA replication is essential for the initiation of meiosis, as errors at this stage can have downstream effects on all subsequent meiotic processes [32]. We previously showed that *atz‐1(ok3406)* mutants are hypersensitive to replication stress and exhibit reduced REC‐8 loading, suggesting a defect in early germline DNA replication [14]. To assess DNA synthesis directly, EdU incorporation was used to visualise actively replicating DNA in the distal germline. In wild‐type animals, a strong EdU signal was observed in the mitotic region, whereas *atz‐1(ok3406)* mutants showed a significant reduction in EdU incorporation (Fig. 3A). This finding indicates that DNA replication is impaired in the absence of ATZ‐1.

Impaired replication at this stage is likely to result in replication stress and activation of checkpoint pathways, which may contribute to the delayed meiotic progression and chromosomal abnormalities observed in *atz‐1* mutants. To determine whether these defects involve cell‐cycle checkpoint pathways, we examined genetic interactions between *atz‐1* and the checkpoint kinases *chk‐1* and *chk‐2*. Knockdown of *chk‐1* in *atz‐1(ok3406)* mutants resulted in significantly increased embryonic lethality compared to wild‐type animals (Fig. 3B). In contrast, knockdown of *chk‐2* did not produce a comparable effect. These findings indicate that *atz‐1* mutants are specifically sensitive to disruption of S‐phase checkpoint function, supporting a role for ATZ‐1 in DNA replication‐associated processes.

## Discussion

Successful progression through meiosis I is critical for establishing the foundation required for normal germ cell maturation. In this study, we show that *atz‐1* is required for maintaining proper meiotic timing and germline organisation, with loss of *atz‐1* resulting in endomitotic oocytes, defects in sperm containment, and extension of the pachytene region. Importantly, these phenotypes are accompanied by a reduction in DNA synthesis and enhanced sensitivity to *chk‐1* knockdown, collectively suggesting that ATZ‐1 functions during early events associated with premeiotic S‐phase.

The reduction of EdU incorporation observed in *atz‐1(ok3406)* mutants suggests that DNA replication is impaired in the distal germline. This defect is likely to have downstream consequences for meiotic progression, as incomplete or erroneous replication can lead to replication stress, activation of checkpoint pathways, and delays in cell‐cycle progression [33, 34, 35]. Replication stress is a well‐established trigger of ATR–CHK1 (ATL‐1‐CHK‐1 in *C. elegans*) signalling, which acts to stabilise replication forks and prevent collapse [36, 37, 38, 39]. Therefore, extension of the pachytene region observed in *atz‐1* mutants is consistent with activation of the pachytene checkpoint, which monitors homologous chromosome pairing and recombination fidelity [11, 12, 40, 41, 42]. In this context, the chromosomal abnormalities observed in diakinetic oocytes may arise as a downstream consequence of earlier defects in DNA replication and checkpoint activation.

Our previous work demonstrated that *atz‐1* mutants exhibit reduced loading of the cohesin protein REC‐8, which is essential for sister chromatid cohesion during meiosis [43, 44, 45, 46]. Cohesin is required not only for chromosome segregation but also for facilitating homologous recombination and synapsis [47, 48, 49, 50]. Defects in cohesin loading impair homologous chromosome pairing and recombination, leading to checkpoint activation and delayed meiotic progression [11, 51]. It is therefore likely that the phenotypes observed in *atz‐1* mutants reflect a combination of impaired DNA replication and defective chromosome organisation, both of which contribute to altered meiotic timing.

The genetic interaction between *atz‐1* and *chk‐1* provides further support for a role of ATZ‐1 in S‐phase‐associated processes. Knockdown of *chk‐1* in *atz‐1* mutants resulted in marked embryonic lethality and a reduced brood size, whereas knockdown of *chk‐2* did not produce a comparable effect. Given that CHK‐1 is primarily involved in regulating DNA replication, stabilising replication forks, and coordinating the intra‐S checkpoint [34, 36, 38], this result suggests that ATZ‐1 functions either upstream of or in parallel with CHK‐1‐dependent pathways. The enhanced sensitivity to *chk‐1* depletion is consistent with a model in which loss of ATZ‐1 compromises replication fork stability, rendering cells more dependent on CHK‐1‐mediated checkpoint control. Failure to properly regulate replication fork progression can result in fork stalling or collapse, leading to the accumulation of DNA damage and genomic instability [33, 52, 53, 54]. The reduced DNA synthesis observed in *atz‐1* mutants, together with its previously reported hypersensitivity to replication stress, supports the idea that ATZ‐1 contributes to maintaining replication fork integrity. In this context, defects in replication during premeiotic S‐phase would propagate through subsequent stages of meiosis, ultimately leading to the chromosomal abnormalities and endomitotic oocytes observed in *atz‐1* mutants.

ATZ‐1 was initially proposed to function as a putative nuclear myosin, raising the possibility that it may act as a motor protein involved in chromosome dynamics or nuclear organisation. While the absence of canonical myosin domains argues against a classical motor function, nuclear myosin‐like proteins have been implicated in chromatin organisation, transcriptional regulation, and DNA repair processes [55, 56, 57, 58]. It remains possible that ATZ‐1 contributes to the spatial organisation of replication or repair machinery within the nucleus, thereby influencing genome stability. Due to this, determining whether ATZ‐1 directly interacts with components of the replication machinery or checkpoint pathways will be of considerable interest.

In conclusion, our findings show that *atz‐1* influences cell‐cycle function in the germline, potentially with a role in premeiotic S‐phase processes. Our previously reported findings where the absence of *atz‐1* correlates with errors in DNA replication complement these new findings of downstream abnormalities in meiotic timing and germ cell development in *atz‐1* deletion mutants. Overall, this study provides new insights into how early disruptions in genome maintenance can impact reproductive success and downstream meiotic function.

## Conflict of interest

The authors declare no conflicts of interest.

## Author contributions

GMD conceived and designed the project. GMD, TEG, LWG, NHW, FE, and PRG acquired the data. GMD, LWG, NHW, and TEG analysed the data. GMD and TEG wrote the manuscript.

## Supporting information


**Fig. S1.** Knockdown of *atz‐1* phenocopies *atz‐1(ok3406)* mutants.

## Data Availability

The data that support the findings of this study are available from the corresponding author [gmdavis@swinburne.edu.au] upon reasonable request.
