# Supplementary material for: ATZ‐1 promotes DNA replication efficiency to maintain normal meiotic function
Source: FEBS Open Bio. 2026 Jul 9:10.1002/2211-5463.70300. Online ahead of print. doi: 10.1002/2211-5463.70300 (PMC13398775; doi:10.1002/2211-5463.70300)
Supplement: Supplementary file 1 — Fig. S1. Knockdown of atz‐1 phenocopies atz‐1(ok3406) mutants. [file FEB4-9999-0-s001.docx]

**Appendix:**

**
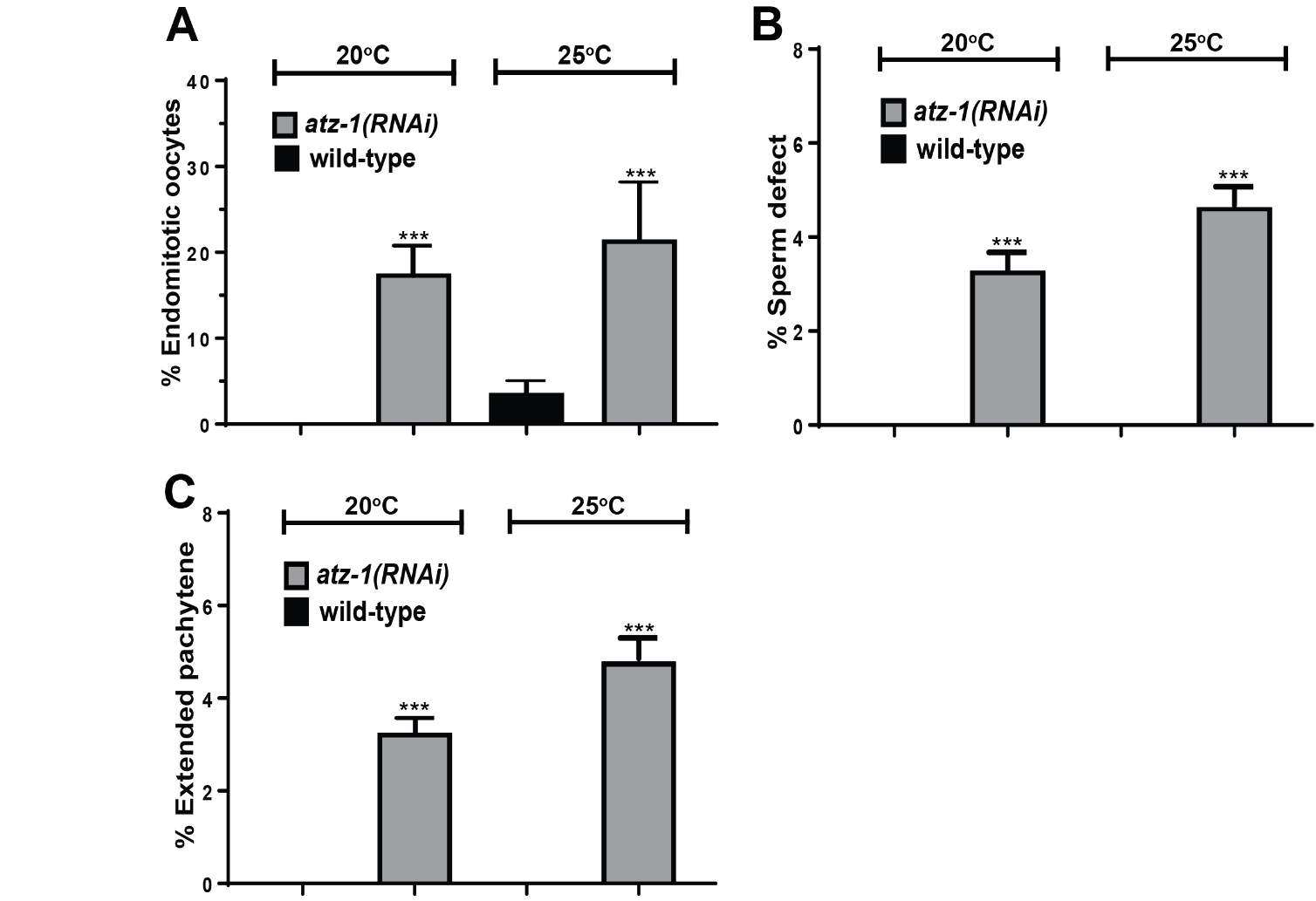
**

**Figure S1: Knockdown of *atz-1* phenocopies *atz-1(ok3406)* mutants:**

RNAi of *atz-1* results in comparable phenotypes to deletion of *atz-1.* Comparable, yet less severe defects observed are the presence of endomitotic oocytes (A) n=100, A sperm containment defect (B) n= 150, and extended pachytene region (C) n=150. Error bars represent standard error of mean (SEM). *** P<0.001 (student t-test).
